# Polymer nanoparticles from low-energy nanoemulsions for biomedical applications

**DOI:** 10.3762/bjnano.14.29

**Published:** 2023-03-13

**Authors:** Santiago Grijalvo, Carlos Rodriguez-Abreu

**Affiliations:** 1 CIBER-BBN, ISCIII, Jordi Girona 18–26, 08034 Barcelona, Spain; 2 Instituto de Quimica Avanzada de Cataluña (IQAC), CSIC, Jordi Girona 18–26, 08034 Barcelona, Spain

**Keywords:** ethyl cellulose, nanoemulsions, nanomedicine, phase inversion composition (PIC) method, PLGA, polymer nanoparticles, polyuria, polyurethane, surfactants

## Abstract

The formulation of nanoemulsions by low-energy strategies, particularly by the phase inversion composition method, and the use of these nanoemulsions as templates for the preparation of polymer nanoparticles for biomedical applications are reviewed. The methods of preparation, nature of the components in the formulation, and their impact on the physicochemical properties, drug loading, and drug release are discussed. We highlight the utilization of ethyl cellulose, poly(lactic-*co*-glycolic acid), and polyurethane/polyurea in the field of nanomedicine as potential drug delivery systems. Advances are still needed to achieve better control over size distribution, nanoparticle concentration, surface functionalization, and the type of polymers that can be processed.

## Review

### 1 Introduction

The field of nanomedicine has yielded several relevant advancements since its beginnings in the early 2000s. The dissolution kinetics of poorly soluble drugs have been improved by the production of drug nanocrystals, enabling continuous drug release. Lipid molecular structures have been manipulated at the nanoscale to escape from endosomes. Notably, lipid nanoparticles enabled the remarkably fast development of mRNA vaccines against COVID-19. Still, there is much to be done to reach the final goal of developing formulations that can deliver drugs at preset rates and periods of time to specific targets [1]. To this end, nanocarriers need to be engineered to add functionalities, both in their cores and at their surfaces. This includes therapeutic drugs and genes, targeting moieties, performance enhancers (e.g., for barrier penetration and to avoid opsonization), and imaging agents [2–3]. Core and matrix of the nanoparticles should be biocompatible, preferentially biodegradable, and with the capacity for proper encapsulation and release of the drug payload. It is also desired that the matrix surface contains reactive or charged groups that facilitate functionalization by covalent or electrostatic bonding.

Herein, we review research on the fabrication of polymer nanoparticles from low-energy nanoemulsions, focusing on phase inversion composition. We particularly emphasize their biomedical applications as drug carriers.

### 2 Nanoemulsions

Nanoemulsions are constituted by nanoscale droplets (20–200 nm) dispersed in a continuous liquid phase. They are out-of-equilibrium nanocolloids in which phase separation is expected from thermodynamics, but is delayed by the presence of surfactants adsorbed on the droplets surface. Accordingly, nanoemulsion formation depends on the way the sample is prepared, for example, on the order of component addition or on the thermal history. Nanoemulsions are not to be confused with microemulsions, which are equilibrium systems with thermodynamic stability [4]. Because of their very small drop size, the main mechanism for nanoemulsion destabilization is commonly attributed to Ostwald ripening. Nanoemulsions can be formulated to contain oily (hydrophobic) droplets in a continuous aqueous phase, that is, as in oil-in-water (O/W) nanoemulsions or aqueous droplets in a continuous oily (hydrophobic) phase as in water-in-oil (W/O) nanoemulsions. To form an emulsion, the liquid to be dispersed should be first fractionated into droplets. This process implies an increase in specific surface area *A* with an associated theoretical energy penalty (*E*_t_) expressed as


[1] $$E_{\text{t}}=\gamma \text{d}A=\frac{6\gamma \phi }{d_{\text{E}}},$$

where γ is the O/W interfacial tension, ϕ is the volume fraction of the dispersed phase, and *d*_E_ is the droplet diameter in the emulsion. In the so-called high-energy methods (also called work-based methods [5]), this energy is supplied by external mechanical means, such as in high-pressure homogenizers or from ultrasound devices, with high dissipation (mostly in the form of heat) and, therefore, low energy efficiencies. In contrast, the so-called low-energy methods (also called thermodynamic methods [5]) overcome this energy barrier by producing low interfacial tensions, changes in the surfactant layer curvature, or gradients of chemical potential between the phases. Herein, we focus on nanoemulsification by low-energy methods.

**Figure 1:**
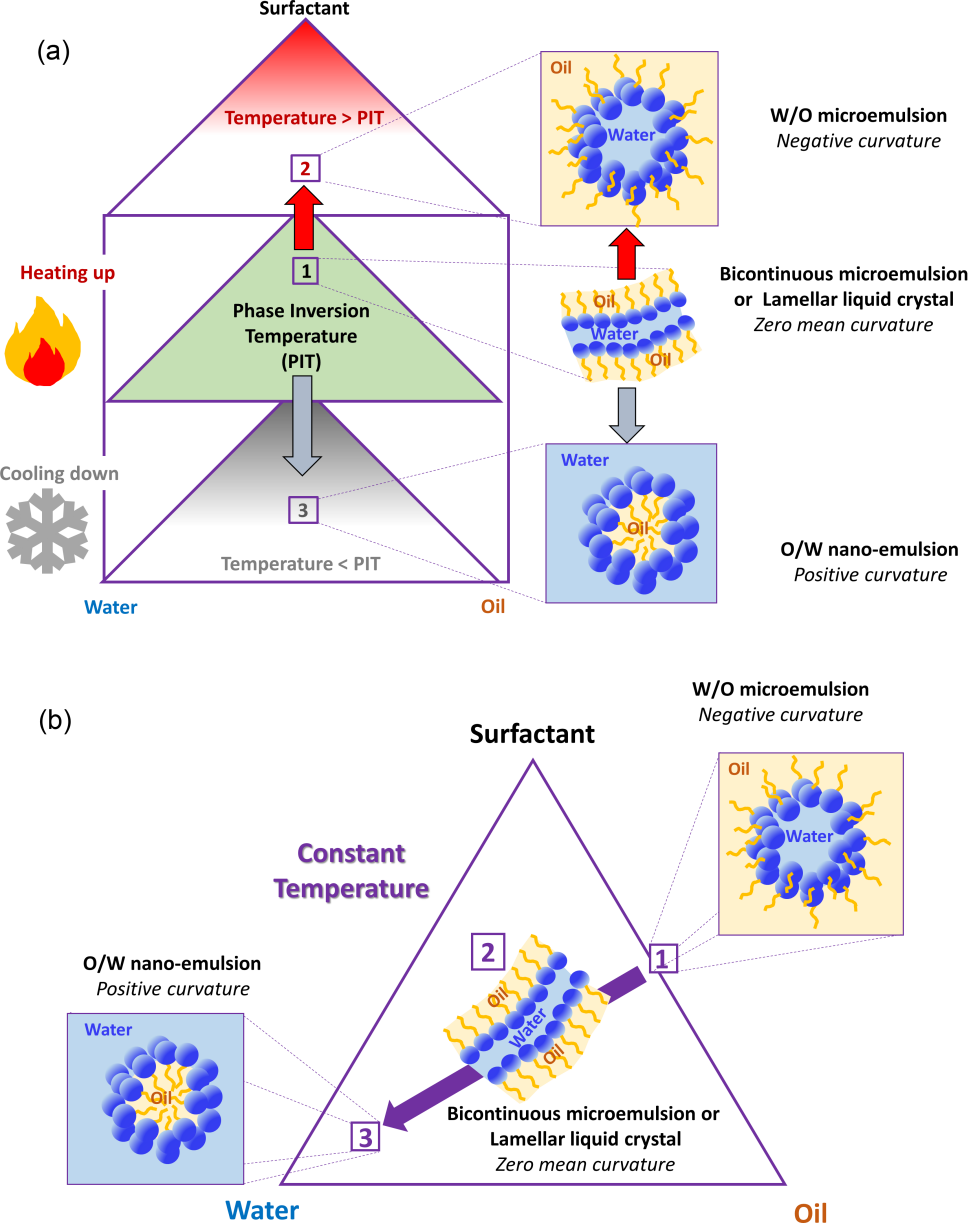
Schematics of the structural and curvature changes during (a) PIT and (b) PIC nanoemulsification.

The two main emulsification processes that involve phase inversion are based on the phase inversion temperature (PIT) or the phase inversion composition (PIC). The PIT method [6–8] applies to nonionic ethoxylated surfactants that change from water-soluble (hydrophilic) to oil-soluble (hydrophobic) with increasing temperature [9]. At low temperature, that is, below the so-called hydrophilic/lipophilic balance (HLB) temperature (*T*_HLB_), the poly(oxyethylene) (POE) chain is highly hydrated, and therefore these surfactants self-assemble at interfaces with positive curvature and are able to form O/W droplets. At temperatures higher than *T*_HLB_, the curvature of the surface interfacial layer is negative (POE chain scarcely hydrated), and thus W/O droplets are formed (Figure 1a). The change in the sign of surface curvature at *T*_HLB_ (also called transitional phase inversion) is associated with extremely low interfacial tensions that promote the formation of very small droplets. However, since the colloidal stability is very low, the systems need to be quenched to lower temperatures to obtain kinetically stable nanoemulsions [10–11].

In contrast, the PIC method (also called emulsion inversion point method) [12–13] proceeds at constant temperature (Figure 1b). Here, the change in the curvature of the surfactant layer from negative to positive or vice versa is driven by the addition of water (which increases POE hydration) to a mixture of oil + surfactant to produce an O/W nanoemulsion [14]. Under continued dilution, the system passes through structures with zero curvature, such as bicontinuous microemulsions or flexible lamellar liquid crystals [15]. The PIC method is amenable for scaling up since it mainly involves a simple dilution process and is suitable for components that cannot withstand high temperatures. Moreover, the composition-driven phase inversion can occur using nonionic or ionic surfactants, which adds flexibility to the process.

Low-energy nanoemulsions can also be produced by processes that do not involve any phase inversion (i.e., any change in the sign of surfactant curvature), such as self-emulsification or spontaneous emulsification [16]. Here, one component present in the oil phase diffuses into the aqueous phase, resulting in the formation of metastable oily droplets by local supersaturation produced near the interface followed by oil nucleation. The ouzo effect is an example of this phenomenon, in which water is further added to a homogeneous solution composed of oil, a short-chain alcohol, and water (without surfactant) [17].

Assuming that all surfactant molecules are adsorbed at the O/W interface, the diameter of the nanoemulsion droplets (excluding the hydrated surfactant layer) can be roughly estimated by [18]:


[2] $$d_{\text{E}}=\frac{6M_{\text{s}}}{a_{\text{s}}N_{\text{A}}}\left(\frac{R_{\text{os}}}{\rho_{\text{o}}}+\frac{v_{\text{L}}}{M_{\text{s}}}\right),$$

where *M*_s_ is the surfactant molecular weight, *a*_s_ is the area occupied per surfactant molecule at the O/W interface, *N*_A_ is Avogadro’s number, ρ_0_ is the density of the oil phase, *R*_os_ is the oil/surfactant mass ratio, and *v*_L_ is the molar volume of the hydrophobic part of the surfactant molecule. Usually, the experimentally measured nanoemulsion droplet sizes are larger than those calculated by Equation 2 [19–20]. This may indicate that not all surfactant molecules are adsorbed at the O/W interface, that is, the actual value of *R*_os_ in the droplets is higher than expected. Excess (non-adsorbed) surfactant can be in the form of micelles that coexist with the nanoemulsion droplets. The kinetics of surfactant adsorption during droplet formation, droplet coarsening, as well as uncertainties in the estimation of *a*_s_ could also explain the divergences between calculated and experimental values of *d*_E_.

Nanoemulsions are versatile templates for the preparation of polymer nanoparticles [21]. To formulate nanoemulsions of predefined aqueous and solvent–polymer or monomer (oil) phases, it is crucial to choose the right surfactant. Also, a careful study of the phase behavior of surfactant/water/oil systems through the use of phase diagrams is usually needed. For biomedical applications, this is further complicated by the fact that the list of available components is restricted by regulations. The most straightforward strategy to obtain polymer nanoparticles from nanoemulsions is to formulate a hydrophobic (oil) phase with the polymer of choice dissolved in a volatile solvent. Then, the volatile solvent can be evaporated from the nanoemulsion droplets under vacuum (that is, at relatively low temperature), and polymer nanoparticles are obtained in the suspension. It is to be noted that the solvent can also be removed by selective diffusion [22].

The emulsion solvent evaporation method enables the use of biocompatible polymers, thermolabile compounds, and low-toxicity surfactants. The nanoparticle size can be tuned by formulation parameters and since the nanoparticles are directly obtained as an aqueous dispersion, it facilitates surface functionalization and substance encapsulation [23]. Nanoparticles obtained from nanoemulsions are usually a few tens of nanometers in size and are smaller than the nanoemulsion droplets. In one example, the nanoemulsion droplet templates had a diameter *d*_E_ = 50 ± 12 nm (estimated by cryo-TEM), whereas the diameter of the derived PLGA nanoparticles (*d*_P_) was 25 ± 7 nm as estimated from TEM [24], which gives a *d*_P_/*d*_E_ ratio of 0.5. Higher *d*_P_/*d*_E_ ratios were found by dynamic light scattering, especially at low polymer concentrations (note that the solubility of polymers in the used solvents rarely exceeds 10 wt %). Experimental *d*_P_/*d*_E_ ratios are above of those expected if the nanoparticles were to be obtained with a density equal to that of the polymer in the bulk, that is, by applying the formula


[3] $$d_{\text{P}}/d_{\text{E}}={\left(\frac{m_{\text{P}}}{m_{\text{T}}}\frac{\rho_{\text{T}}}{\rho_{\text{P}}}\right)}^{1/3},$$

where *m*_P_*/m*_T_ is the polymer/solvent mass ratio and ρ_T_/ρ_P_ is the solvent/polymer bulk density ratio (this equation assumes that each droplet generates one single nanoparticle). In other words, the polymer nanoparticles seem to be, in general, less dense than in the bulk, which could be attributed to porosity generated during solvent evaporation. Conventional emulsification methods generally yield nanoparticles with sizes larger than those described above. For example, emulsions prepared with poly(vinylalcohol) (PVA) as stabilizer produce PLGA polymer microparticles with a size of several tens of micrometers after extraction or evaporation of the solvent [25]. The droplets of nanoemulsions produced by high-energy methods, for example, by sonication [26], show sizes larger than those from PIC method, and therefore the derived nanoparticles are also larger [27–29].

The preparation of various types of polymer nanoparticles by polymerization through phase inversion has been reported [30–35]*.* A phase behavior study is commonly needed to find the appropriate surfactant for a given monomer, as well as the conditions for phase inversion in terms of temperature and/or composition. This review is centered on polymers of biomedical interest mainly prepared by nanoemulsification/evaporation.

### 3 Preparation of polymer nanoparticles by low-energy emulsification

#### 3.1 Ethyl cellulose nanoparticles

Ethyl cellulose is a biocompatible and FDA-approved polymer (a chemical substance generally recognized as safe) widely applied within the biomedical and pharmaceutical industry [36–37]. One of the earliest reports on the preparation of ethyl cellulose nanoparticles from low-energy nanoemulsions is that of Spermath and Magdassi [38]. They prepared a PIC nanoemulsion by addition of a 10 mM NaCl solution to ethyl cellulose dissolved in toluene. A mixture of sorbitan monolaurate (Span^®^ 20) plus decaglycerol monolaurate (DML) was used as surfactant. The nanoemulsions had an average droplet size between 128 and 279 nm. After toluene evaporation, nanoparticles with sizes in the range of 50–100 nm were obtained. Oil-in-water nanoemulsion and evaporation protocols allowed Generalova et al. [39] to prepare ethyl cellulose nanoparticles containing both hydrophobic and hydrophilic fluorescent nanocrystals. Size measurements showed that nanoparticles were in the range of 128–132 nm. Additionally, the corresponding fluorescence-labeled nanoparticles were functionalized with the monoclonal antibody F19 via carbodiimide conjugation. This approach enabled the use of ethyl cellulose nanoparticles in bioanalytical applications with the aim to detect *Yersinia* pestis from direct agglutination tests. Irradiation of nanoparticles under UV light favored flocculation of the nanoparticles with an analyte detection limit of 10 μg/mL.

Calderó et al. [40] prepared PIC nanoemulsions at room temperature using poly(oxyethylene)(4) sorbitan monolaurate as surfactant. The hydrophobic phase dispersed in the droplets was constituted by 10% ethyl cellulose dissolved in ethyl acetate. This solvent is less toxic than commonly used aromatic compounds. Kinetically stable O/W nanoemulsions were obtained at oil/surfactant (O/S) mass ratios between 30/70 and 70/30 and water contents above 40 wt %. The light transmittance of the sample increased with water content and decreased with O/S ratio. The hydrodynamic diameter of the nanoemulsion droplets as measured by dynamic light scattering (DLS) was ca. 200 nm. It should be pointed out that ethyl acetate is a polar oil, and that the ethyl acetate/water interfacial tension is low (6.8 mN/m [41]). Usually, the excess interfacial concentration of the surfactant is lower for polar oils with low interfacial tension than for nonpolar oils [42], which is one of the causes that the actual droplet sizes are higher than those estimated from Equation 2. The ethyl cellulose nanoparticles obtained from these nanoemulsions after ethyl acetate evaporation showed an average diameter between 29 and 44 nm as determined from TEM images. The diameter appeared to increase with the O/S ratio (in qualitative agreement with Equation 2 and Equation 3), but was much smaller than that of the nanodroplet templates as a result of solvent evaporation.

**Figure 2:**
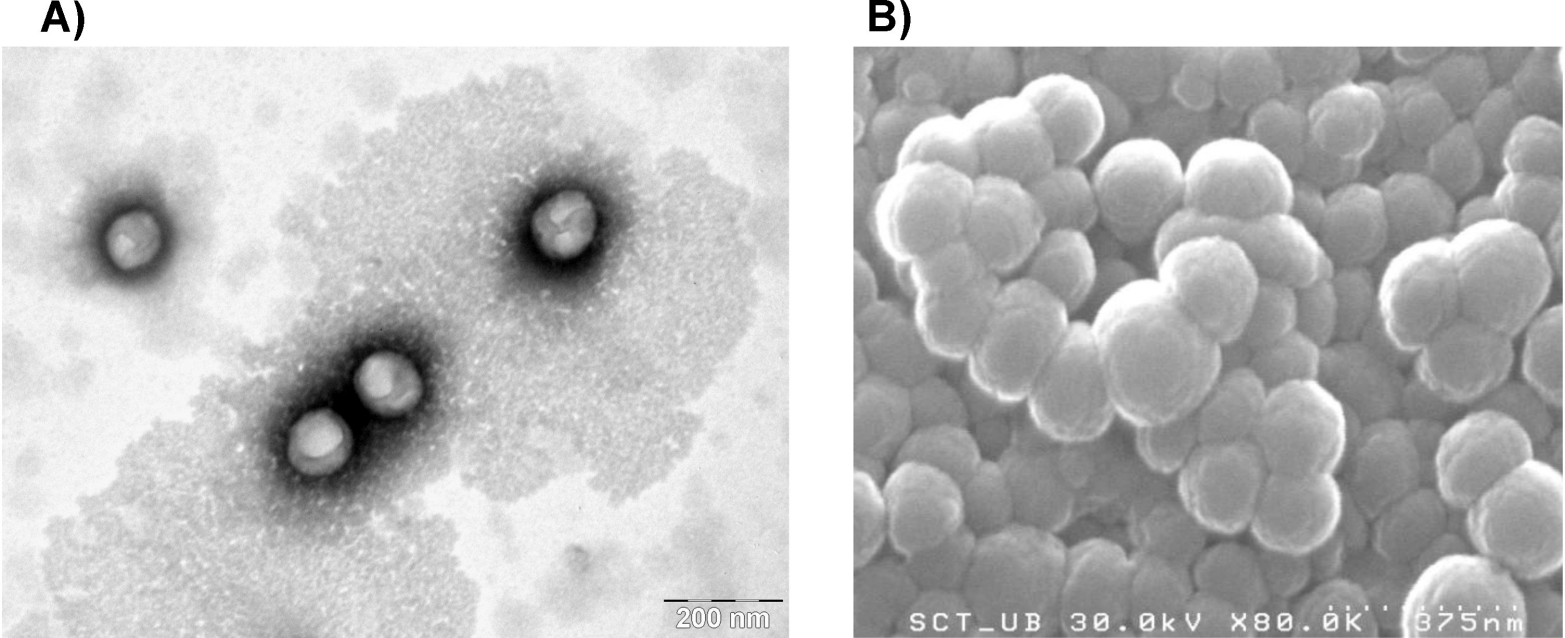
(A) TEM and (B) SEM images of ethyl cellulose nanoparticles obtained from nanoemulsions with an O/S (ethyl acetate/poly(oxyethylene)(10) oleyl ether) ratio of 70/30 and 90 wt % of water. The ethyl acetate phase contained 4 wt % of ethyl cellulose. Figure 2 was reprinted from [43], Colloids and Surfaces B: Biointerfaces, vol. 145, by G. Calderó; R. Montes; M. Llinàs; M. J. García-Celma; M. Porras; C. Solans, “Studies on the formation of polymeric nanoemulsions obtained via low-energy emulsification and their use as templates for drug delivery nanoparticle dispersions”, pages 922-931, Copyright (2016), with permission from Elsevier. This content is not subject to CC BY 4.0.

Nanoemulsions were also formulated at room temperature from poly(oxyethylene)(10) oleyl ether [43] containing ethyl cellulose (4%) dissolved in ethyl acetate. Nanoemulsions were obtained after phase inversion (as confirmed by electrical conductivity measurements) at O/S ratios between 70/30 and 80/20 and water contents above 87 wt %, with droplet sizes in the 180–190 nm range. The derived ethyl cellulose nanoparticles had a size between 107 and 161 nm as estimated by TEM and SEM (Figure 2). Dexamethasone (DXM), a steroid with potent anti-inflammatory and immunosuppressant activity, was encapsulated in these ethyl cellulose nanoparticles by dissolving it in the hydrophobic droplet phase of the starting nanoemulsions. The drug release from the nanoparticles appeared to follow a coupled diffusion/relaxation model.

Nanoemulsions containing ethyl cellulose have also been prepared at room temperature by the PIC method from mixtures of nonionic and cationic surfactants [44]. More specifically, in the system water/(ricinoleamidopropyltrimonium methosulfate/Cremophor® WO7)/(6% ethyl cellulose in ethyl acetate), nanoemulsions are formed at O/S ratios between 55/45 and 75/25 and above 88 wt % water (Cremophor^®^ WO is an ethoxylated castor oil) [45]. The hydrodynamic droplet diameters were between 120 and 170 nm (at 90 wt % water) depending on the O/S ratio. The diameters of the derived ethyl cellulose nanoparticles were in a similar range (100–200 nm), depending on the ratio between cationic and nonionic surfactant.

Nanoemulsions prepared from the water/(ricinoleamidopropyltrimonium methosulfate/sorbitan monooleate = 1:1)/(6 wt % EC10 in ethyl acetate) system at 25 °C [46] were less stable than those prepared from Kolliphor^®^ EL and showed larger droplet sizes (between 230 and 300 nm) but still could be used to prepare ethyl cellulose nanoparticles with average sizes between 220 and 250 nm as measured by TEM.

A mixture of ricinoleamidopropyltrimonium methosulfate and Kolliphor^®^ EL (ethoxylated castor oil) was also used to prepare O/W nanoemulsions with HEPES buffer solution as continuous phase and a 6 wt % solution of ethylcelullose in ethyl acetate as the dispersed phase [47]. Nanoemulsions formed at O/S ratios between 45/55 and 90/10, above 35 wt % HEPES solution content. The nanoemulsion droplets showed an average hydrodynamic diameter between 100 and 120 nm, which remained practically constant over at least three weeks. The ethylcelullose nanoparticles obtained after solvent evaporation displayed a positively charged surface (zeta potential about +22 mV) and their TEM average size was about 40 nm. The nanoparticles were complexed with an antisense phosphorothioate oligonucleotide targeting *Renilla* luciferase mRNA. Gene inhibition showed an optimum efficiency (40%) at a given nanoparticle/antisense oligonucleotide ratio, which is promising for in vitro cell transfection.

When water was replaced with PBS (0.16 M) for the PIC preparation of nanoemulsions starting from ricinoleamidopropyltrimonium methosulfate + Kolliphor^®^ EL + 6 wt % ethyl cellulose in ethyl acetate, an increase in the area of the nanoemulsion region in the phase diagram was observed [47]. The average nanoparticle size also slightly decreased (from 45 to 40 nm) when water was replaced by HEPES solution as the aqueous phase in the nanoemulsion. These nanoparticles were complexed with folic acid and showed low hemolytic activity (below 5%).

The characteristics of the reported PIC nanoemulsions and derived ethyl cellulose nanoparticles are summarized in Table 1.

**Table 1:** Formulation parameters of starting nanoemulsions, droplet size, and size of derived ethyl cellulose nanoparticles prepared by the PIC method. *d*_E_ and *d*_P_ are the diameters of nanoemulsion droplets and nanoparticles, respectively.

Surfactant	Aqueous phase	Oil (organic) phase	O/S ratio	*d*_E_ (nm)^a^	*d*_P_ (nm)^b^	Ref.

poly(oxyethylene)(4) sorbitan monolaurate	water (ca. 90 wt %)	10 wt % ethyl cellulose in ethyl acetate	30/70 to 70/30	ca. 200	29–44	[40]
poly(oxyethylene)(10) oleyl ether	water (ca. 90 wt %)	4 wt % ethyl cellulose in ethyl acetate	70/30 to 80/20	180–190	107–161	[43]
ricinoleamidopropyltrimonium methosulfate and Cremophor^®^ WO7	water (ca. 90 wt %)	6 wt % ethyl cellulose in ethyl acetate	55/45 to 75/25	120–170	no TEM data^c^	[45]
ricinoleamidopropyltrimonium methosulfate/sorbitan monooleate	water (ca. 90 wt %)	6 wt % ethyl cellulose in ethyl acetate	50/50 to 70/30	230–300	220–250	[46]
ricinoleamidopropyltrimonium methosulfate and Kolliphor^®^ EL	HEPES 20 mM (ca. 95 wt %)	6 wt % ethyl cellulose in ethyl acetate	45/55 to 90/10	100–120	ca. 40	[47]

^a^from DLS; ^b^from TEM; ^c^average nanoparticle diameters from DLS were 100–200 nm.

#### 3.2 Poly(lactic-*co*-glycolic acid) nanoparticles

Poly(lactic-*co*-glycolic acid) (PLGA) is a biodegradable polymer that decomposes by hydrolysis into non-toxic and easily metabolized monomers, namely lactic and glycolic acid. It is approved by FDA and EMA [23,48]. Biodegradable and biocompatible PLGA nanoparticles find uses as carriers for drugs, peptides, proteins, vaccines, and nucleotides [2]. In spite of biodegradability and biocompatibility, some studies have also demonstrated a certain concentration-dependent toxicologic profile including a mild inflammatory response after treatment with PLGA nanoparticles [49]. Some authors have suggested that the inherent cytotoxicity of PLGA may come from the accumulation of the degraded polymer components leading to changes in osmolality of the culture medium [50]. Other in vitro studies also reported that the size of PLGA nanoparticles had a significant impact on cytotoxicity against BEAS-2B and RAW264.7 cells [51].

Several PLGA-based products have been released to the pharmaceutical market (e.g., Lupron Depot^®^) [1,25,29,52]. Fornaguera et al. [53] produced nanoemulsions from a mixture of poly(oxyethylene)(20) sorbitan monooleate (Polysorbate 80) as surfactant, a 4 wt % solution of PLGA in ethanol/ethyl acetate as oil (organic) phase, and a PBS solution as aqueous phase. Nanoemulsions were formed for O/S ratios between 50/50 and 90/10, with hydrodynamic diameters around 80 nm. PLGA nanoparticles with a hydrodynamic diameter of ca. 60 nm were produced from the nanoemulsions by ethyl acetate evaporation. These nanoparticles featured a negatively charged surface (zeta potential between −30 and −40 mV) resulting from the terminal carboxyl groups of PLGA. The drug loperamide was solubilized in the nanoemulsion droplets so that loperamide-loaded PLGA nanoparticles with a hydrodynamic diameter of ca. 200 nm were obtained upon solvent removal. High colloidal stability (longer than three months without sedimentation), high encapsulation efficiency (>99%), slow drug release (only 15% of the drug after five days), and low cytotoxicity against HeLa cells (cell viability > 80%) were observed. In vivo tests showed that the PLGA nanoparticles functionalized with 8D3 antibody were able to cross the blood–brain barrier (BBB) as demonstrated by the analgesic effect of encapsulated loperamide on mice.

**Figure 3:**
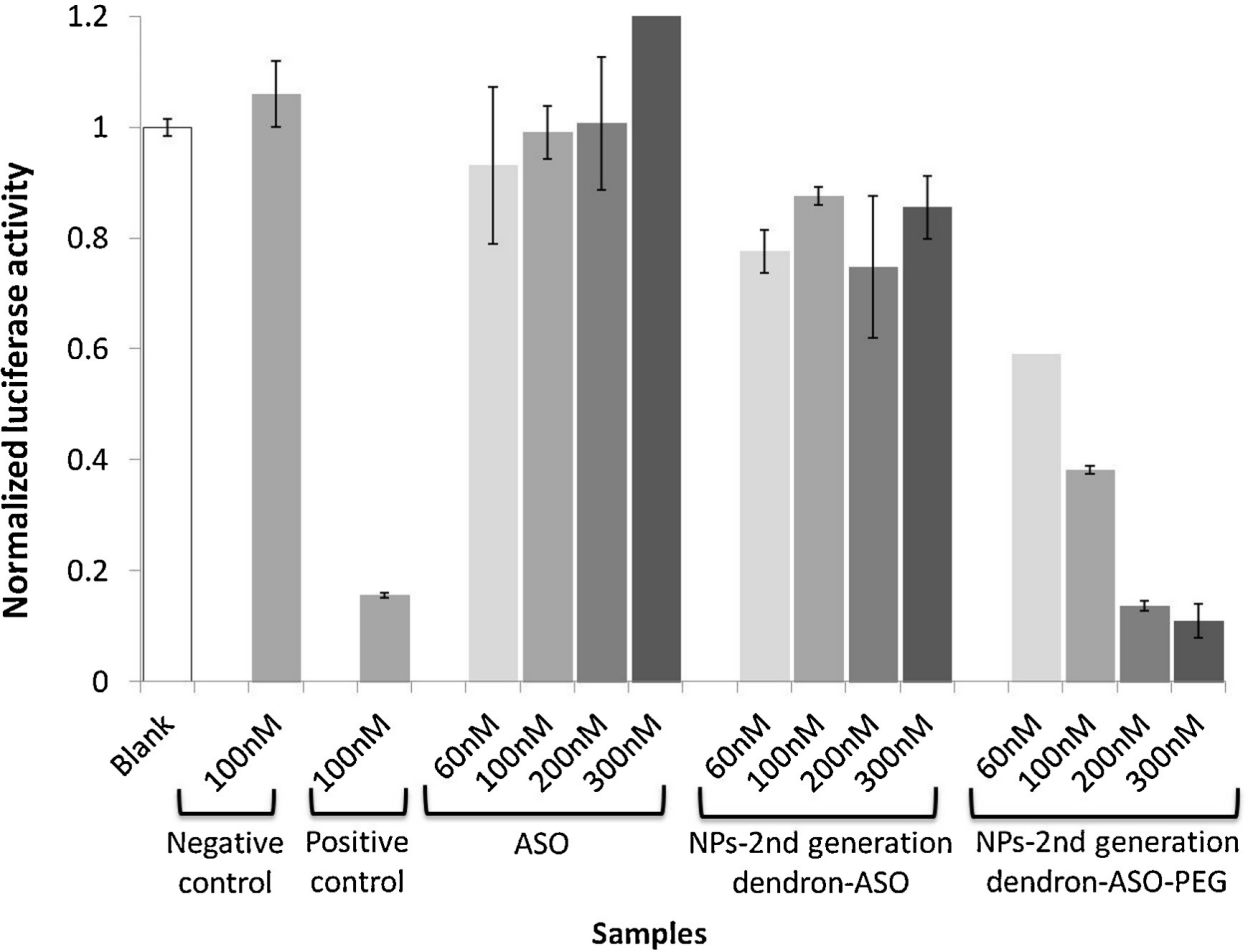
Luciferase activity inhibition (%) for complexes formulated with PLGA nanoparticles from nanoemulsions and a second-generation dendron at a nanoparticle/antisense oligonucleotide ratio of 0.75. Lipofectamine 2000 was used as the positive control and a scrambled antisense oligonucleotide as the negative control. Figure 3 was reprinted from [54], International Journal of Pharmaceutics, vol. 478. issue 1, by C. Fornaguera; S. Grijalvo; M. Galán; E. Fuentes-Paniagua; F. J. de la Mata; R. Gómez; R. Eritja; G. Calderó; C. Solans, “Novel non-viral gene delivery systems composed of carbosilane dendron functionalized nanoparticles prepared from nanoemulsions as non-viral carriers for antisense oligonucleotides”, pages 113-123, Copyright (2014), with permission from Elsevier. This content is not subject to CC BY 4.0.

PLGA nanoparticles prepared using Polysorbate 80 with the same formulation discussed above (diameter ca. 27 nm by TEM) were surface-decorated with carbosilane cationic dendrons via carbodiimide chemistry [54] for conjugation with antisense oligonucleotides (ASO). The conjugated nanoparticles with a hydrodynamic diameter between 80 and 160 nm (depending on dendron generation) showed high colloidal stability and cell viabilities higher than 75% (for concentrations below 100 nM). These nanoparticles displayed high gene silencing efficiencies (up to 90%) targeting *Renilla* luciferase mRNA, particularly when coated with PEG (Figure 3) and did not show hemolysis after incubation with red blood cells.

PLGA nanoparticles were also used to encapsulate therapeutic concentrations of antiinflammatory DXM, initially solubilized in Polysorbate 80 template nanoemulsions [55]. These nanoemulsions formed at O/S ratios between 45/55 and 75/25 and showed hydrodynamic diameters in the 290–326 nm range for a water content of 90%. Their colloidal stability increased with surfactant concentration. The derived PLGA nanoparticles showed average diameters between 109 and 145 nm as determined from TEM, while corresponding average hydrodynamic diameters were in the 264–327 nm range. The DXM entrapment efficiencies (>74%) decreased as the O/S ratio increased, and DXM-loaded PLGA nanoparticles showed dose-dependent cytotoxicity (A549 cell line) and hemolytic response. Encapsulation of DXM in PLGA nanoparticles resulted in slower and sustained release as compared to non-encapsulated DXM.

Nanoemulsions formed in a 0.16 M PBS (W)/Polysorbate 80 (S)/(4 wt % PLGA + 0.1 wt % galantamine in ethyl acetate) (O) system were used as templates to prepare PLGA nanoparticles loaded with galantamine (GAL), a drug to treat neurodegenerative diseases [56]. The nanoparticles displayed an average hydrodynamic diameter of ca. 44 nm with a negative surface charge (ca. −11 mV). The drug encapsulation efficiencies were higher than 98%, although loadings were not enough to achieve therapeutic concentrations. The GAL release from the nanoparticles was slower than that from aqueous GAL solutions and surfactant micelles. Viabilities of HeLa and SH-SY5Y cells incubated with the loaded nanoparticles were close to 100% for GAL concentrations of 0.3 mg/mL. The pharmacological activity of GAL was preserved after encapsulation (as measured by the acetylcholinesterase inhibition test).

PLGA nanoparticles prepared from a 0.16 M PBS (W)/Polysorbate 80 (S)/(4 wt % PLGA in ethyl acetate/ethanol) (O) system were functionalized with a cell-penetrating peptide (Tat) using carbodiimide chemistry [57]. The hydrodynamic diameter ranged from 48 to 65 nm depending on the Tat fraction. The functionalized nanoparticles showed dose-dependent cytotoxicity on Hela and SH-SY5Y cells, and no hemolysis was observed. The presence of Tat on the surface of the nanoparticles enabled cell membrane penetration and uptake in HeLa Cells.

**Figure 4:**
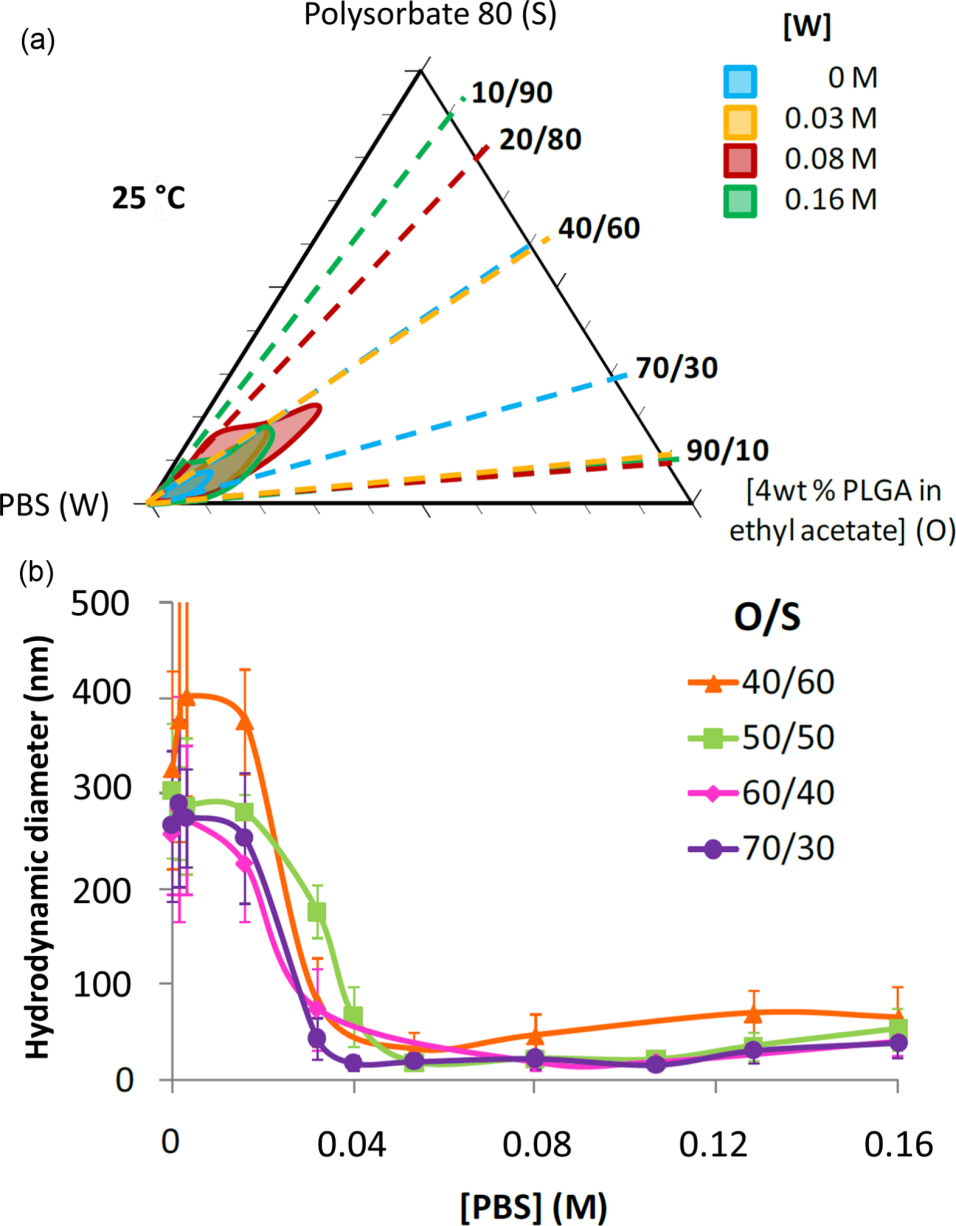
(a) Partial phase diagram of the system PBS/Polysorbate 80/4% PLGA in ethyl acetate. The O/W nanoemulsion regions are shown for various PBS concentrations. (b) Hydrodynamic diameter as function of the PBS concentration for PLGA nanoparticles derived from nanoemulsions prepared with 90 wt % of aqueous phase at different O/S ratios. Figure 4 was used with permission of The Royal Society of Chemistry from [58] (“Electrolytes as a tuning parameter to control nanoemulsion and nanoparticle size” by C. Fornaguera et al., RSC Advances, vol.6, issue 63, © 2016); permission conveyed through Copyright Clearance Center, Inc. This content is not subject to CC BY 4.0.

The effect of electrolytes in the aqueous phase on Polysorbate 80-based PIC nanoemulsions and derived PLGA nanoparticles has been explored [58]. The region for nanoemulsion formation in the phase diagram changes with the electrolyte (PBS) concentration (Figure 4a). There seems to be an optimum value for which this region is the largest, and the colloidal stability is maximum. Note that the presence of electrolyte is expected to reduce Ostwald ripening, especially considering that the solubility of ethyl acetate in water is relatively high (about 80 g/L at 20 °C). The hydrodynamic diameter of the particles decreases sharply (from 300–400 nm to ca. 40 nm) at a given electrolyte concentration (Figure 4b), with a concomitant decrease in the absolute value of the surface zeta potential as a result of charge screening.

Hydrophobic (oleic acid-coated) magnetic nanoparticles have also been incorporated into PLGA nanoparticles prepared from Kolliphor^®^ EL and Polysorbate 80 nanoemulsions [59]. The starting nanoemulsions had an average hydrodynamic droplet diameter of ca. 230 nm for Kolliphor^®^ EL and ca. 125 nm for Polysorbate 80, whereas the size of the resulting hybrid nanoparticles was ca. 40 and 20 nm, respectively, as determined from TEM. The magnetic loading reached up to ca. 20 wt %, and therefore the hybrids could be useful for imaging and magnetic hyperthermia. These particle sizes were smaller than those obtained by high-energy methods (i.e., sonication) [60].

The presence of PLGA in the oil (organic phase) impacts the phase behavior of surfactant systems and thus the phase transitions that take place upon water addition to the oil + surfactant mixture in the PIC method. For example, in the water/Cremophor^®^ EL/PLGA + ethyl acetate system [24], the O/W nanoemulsion region shifts slightly to higher surfactant concentrations (lower O/S ratios) as the PLGA concentration is increased. This seems to be correlated to a concomitant shift of the liquid crystalline phase in the middle of the phase diagram (and also in the middle of the water dilution path). The average hydrodynamic diameter of the nanoemulsion droplets increases from ca. 20 to ca. 140 nm when the PLGA concentration is increased from 0.5 to 4 wt %. However, the droplet size does not change much with time in this concentration range, indicating good colloidal stability, although for the highest concentration, the change in transmittance with time is more noticeable. The size of the derived nanoparticles increases with the PLGA concentration in the ethyl acetate phase, but it is always smaller (ca. 25 nm from TEM) than the precursor nanoemulsion droplets, regardless of the PLGA concentration. DXM can be encapsulated with efficiencies higher than 88% for PLGA concentrations in the 0.5–4 wt % range. The drug release kinetics seems to be slower as the PLGA concentration in the precursor nanoemulsions is increased. Cell viabilities (HeLa cells) were higher than 70% when incubated with non-loaded and DXM-loaded PLGA nanoparticles.

**Figure 5:**
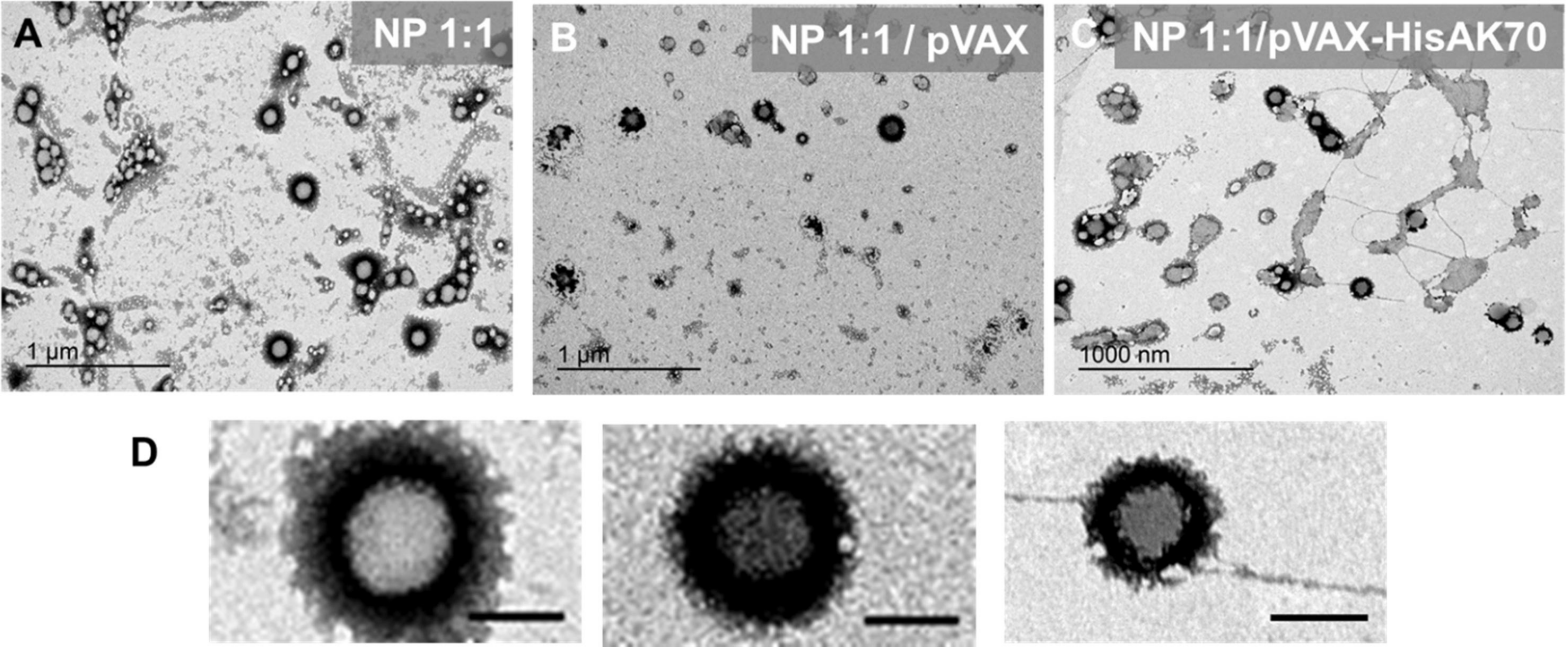
Representative TEM images of negatively stained nanoparticles (NPs) complexed with DNA plasmids (pVAX and pVAX-HisAK70). (A) NP 1:1. (B) NP 1:1/pVAX = 5/1. (C) NP 1:1/pVAX-HisAK70 = 10/1. (D) Zoomed region from each sample (A–C) (all scale bars represent 100 nm). Figure 5 was reprinted from [61], European Polymer Journal, vol. 120, by E. Soler Besumbes; C. Fornaguera; M. Monge; M. J. García-Celma; J. Carrión: C. Solans; A. Dols-Perez, “PLGA cationic nanoparticles, obtained from nanoemulsion templating, as potential DNA vaccines”, article No. 109229, Copyright (2019), with permission from Elsevier. This content is not subject to CC BY 4.0.

PLGA nanoparticles have been also produced from nonionic/cationic surfactant nanoemulsions, specifically in the system water/(ricinoleamidopropyltrimonium methosulfate/Kolliphor^®^ EL)/4% PLGA in ethyl acetate [61]. The nanoparticles displayed a hydrodynamic diameter of ca. 125 nm for an O/S ratio of 70/30, and the size increased with the O/S ratio. The surface charge can be tuned by changing the cationic/nonionic surfactant ratio, that is, the surface becomes positive at a given cationic surfactant concentration and, therefore, the nanoparticles can be complexed with DNA plasmids. This complexation leads to an increase in the hydrodynamic diameter. This increase is larger for plasmids with higher molecular weight. The obtained complexes (Figure 5) with viabilities higher than 70% for HeLa cells are promising candidates for gene therapy (e.g., gene vaccines).

Protein binding on PLGA nanoparticles prepared from nanoemulsions has also been studied [62]. After incubation with human serum, afamin was one of the specific proteins bound to PLGA nanoparticles functionalized with the antibody 8D3. Afamin facilitates the transport of vitamin E to the central nervous system. When the nanoparticles were loaded with a drug, apolipoproteins involved in the transport through the BBB were also identified.

Phytochemicals with antioxidant activity have been encapsulated in PLGA nanoparticles derived from PIC nanoemulsions [63]. These antioxidant-loaded nanoparticles feature hydrodynamic sizes between 71 and 160 nm and encapsulation efficiencies higher than 64%. The colloidal stability of the nanoparticle dispersions was not significantly affected by protein corona formation (upon incubation in FBS). The nanoparticles showed low cytotoxicity for SH-SY5Y cells (viabilities of ca. 100% for nanoparticle concentrations equal or lower than 0.23 mg/mL), high cellular uptake (in SH-SY5Y cells), and dose-dependent antioxidant activity.

The properties of the PIC nanoemulsions and derived PLGA nanoparticles discussed above are summarized in Table 2.

**Table 2:** Formulation parameters of starting nanoemulsions, droplet size and size of derived PLGA nanoparticles prepared by the PIC method. *d*_E_ and *d*_P_ are the diameters of nanoemulsion droplets and nanoparticles, respectively.

Surfactant	Aqueous phase	Oil (organic)phase	O/S ratio	*d*_E_ (nm)^a^	*d*_P_ (nm)^b^	Ref.

polysorbate 80	PBS (0.16 M at 90 wt %)	4 wt % PLGA in ethyl acetate	50/50 to 90/10	ca. 80	ca. 27	[53–54]
polysorbate 80	water (at 90 wt %)	4 wt % PLGA in ethyl acetate + DXM	45/55 to 75/25	290–326	109–145	[55]
Kolliphor^®^ EL	water (at 90 wt %)	6% PLGA in ethyl acetate with 0.5 wt % MNP	60/40	ca. 230	ca. 40	[59]
polysorbate 80	PBS (0.16 M at 90 wt %)	4% PLGA in ethyl acetate with 0.06 wt % MNP	70/30	ca. 125	ca. 20	[59]
Cremophor^®^ EL	water (at 90 wt %)	PLGA in ethyl acetate	55/45 to 70/30	20–140	ca. 25	[24]
ricinoleamidopropyltrimonium methosulfate/Kolliphor^®^ EL	PBS (at 90 wt %)	4% PLGA in ethyl acetate	70/30	ca. 125	ca. 130	[61]

^a^from DLS; ^b^from TEM.

#### 3.3 Polyurethane and polyurea nanoparticles

Polyurethane and polyurea are polymers that can be made biocompatible and display superior physicochemical properties. Therefore, they are attractive for biomedical applications [64–66]. Polyurea nanocapsules have been prepared using the PIT method [67]. The starting nanoemulsions were formulated by dissolving the monomer toluene 2,4-diisocyanate (TDI) in different hydrocarbon oils and then mixing this oil phase with poly(oxyethylene)(10) oleyl ether as surfactant and an aqueous phase containing 10 mM NaCl. The mixtures were heated above the PIT and then quickly cooled down to obtain O/W nanoemulsions. The obtained nanocapsules showed a diameter between 92 and 113 nm, similar to the droplet sizes of nanoemulsion templates.

**Figure 6:**
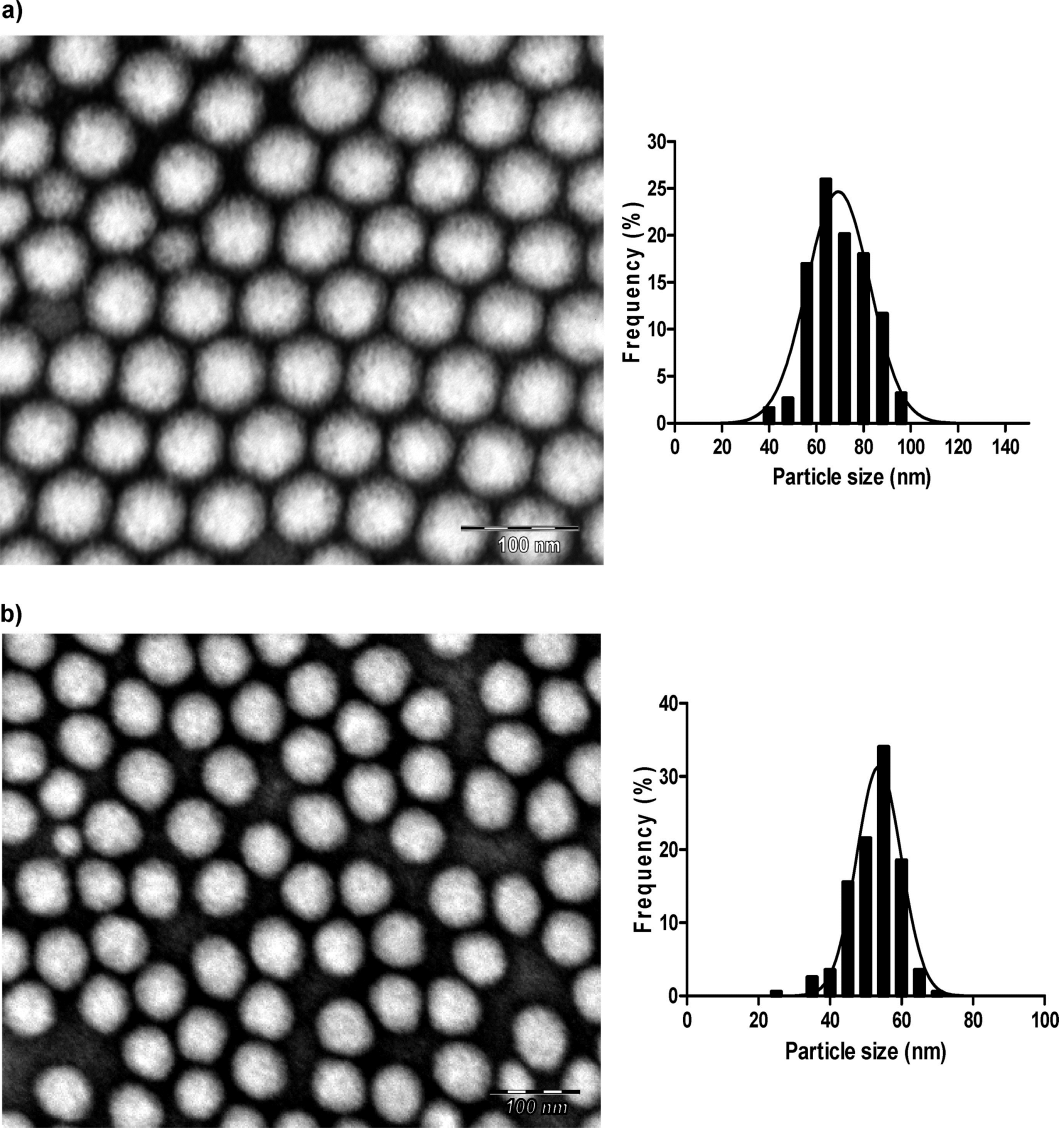
TEM images and corresponding size distributions of (a) PEGylated polyurethane and (b) lysine-coated polyurea nanocapsules obtained from O/W nanoemulsions in an aqueous solution/polysorbate 80/medium chain triglyceride/diisocyanate system at O/S = 10/90 and 90 wt % of aqueous component. Figure 6 was adapted with permission from [68], Copyright 2012 American Chemical Society. This content is not subject to CC BY 4.0.

PIC nanoemulsions have also been used in the synthesis of polyurethane and polyurea from the reaction of poly(ethyleneglycol) or lysine with isophorone diisocyanate (IPDI) [68–69]. Nanoemulsions were prepared using Polysorbate 80 as surfactant, medium-chain triglycerides as the oil phase (O/S ratio from 10/90 to 30/70), and 90 wt % of aqueous phase. The average hydrodynamic diameters of the droplets in those systems were between 15 and 29 nm. The derived polyurethane nanoparticles showed average sizes between 27 and 68 nm, whereas the size of polyurea nanoparticles was in the range of 30–52 nm as measured by TEM (Figure 6). These sizes are much smaller than samples of similar polymers obtained by high-energy emulsification [70–71]. HUVEC cells incubated with nanoparticles synthesized from nanoemulsions at an O/S ratio of 10/90 showed viabilities close to 100%. An increase to an O/S ratio of 20/80 caused a drop in viability of HUVEC cells (higher toxicity) as a result of higher monomer concentration. No hemolytic effect was observed.

## Summary and Outlook

Tailored nanoemulsions with controlled size and colloidal stability can be produced by low-energy methods after careful selection of formulation parameters such as type and concentration of surfactant, oil/surfactant ratio, and ionic strength. The nanoemulsion droplets are versatile templates for the preparation of polymer nanoparticles by the emulsion/solvent evaporation pathway. Particularly, the PIC nanoemulsification method has proven to be very effective for the preparation of ethyl cellulose, PLGA, and polyurethane/polyurea nanoparticles in the form of colloidal suspensions to enable the encapsulation of not only small molecule drugs but also macromolecules. This strategy improved pharmacokinetic properties and biological activities both in vitro and in vivo, showing great promise for the treatment of a vast number of diseases including cancer and neurological diseases. Continuous effort in the field of designing novel polymeric nanoparticle-based formulations might contribute to reduce the existing gap between preclinical and clinical models. This extensive research might overcome the limitations exhibited by conventional therapies, placing drug-containing polymeric nanoparticles as promising therapeutics in the field of nanomedicine.

Future challenges in the preparation of polymer nanoparticles from low-energy nanoemulsions include narrowing size distributions, increasing the concentration of nanoparticles in the as-prepared samples, improving surface functionalization, and expanding the range of polymers that can be processed by this approach.
